# Friction Forces during Sliding of Various Brackets for Malaligned Teeth: An In Vitro Study

**DOI:** 10.1155/2013/871423

**Published:** 2013-02-28

**Authors:** Vito Crincoli, Letizia Perillo, Maria Beatrice Di Bisceglie, Antonio Balsamo, Vitaliano Serpico, Francesco Chiatante, Carmine Pappalettere, Antonio Boccaccio

**Affiliations:** ^1^Department of Dental Sciences and Surgery, Faculty of Medicine and Surgery, University of Bari Aldo Moro, 70124 Bari, Italy; ^2^Department of Dental, Orthodontic and Surgical Disciplines, Second University of Naples, 80138 Naples, Italy; ^3^Department of Mechanics, Mathematics and Management (DMMM), Polytechnic University of Bari, Viale Japigia 182, 70126 Bari, Italy; ^4^Società Cooperativa Sociale e Sanitaria ONLUS “Insieme con Padre Pio”, 74015 Martina Franca, Italy; ^5^European Society for Experimental Mechanics (EuraSEM), Italy

## Abstract

*Aims*. To measure the friction force generated during sliding mechanics with conventional, self-ligating (Damon 3 mx, Smart Clip, and Time 3) and low-friction (Synergy) brackets using different archwire diameters and ligating systems in the presence of apical and buccal malalignments of the canine. *Methods*. An experimental setup reproducing the right buccal segment of the maxillary arch was designed to measure the friction force generated at the bracket/wire and wire/ligature interfaces of different brackets. A complete factorial plan was drawn up and a three-way analysis of variance (ANOVA) was carried out to investigate whether the following factors affect the values of friction force: (i) degree of malalignment, (ii) diameter of the orthodontic wire, and (iii) bracket/ligature combination. Tukey post hoc test was also conducted to evaluate any statistically significant differences between the bracket/ligature combinations analyzed. *Results*. ANOVA showed that all the above factors affect the friction force values. The friction force released during sliding mechanics with conventional brackets is about 5-6times higher than that released with the other investigated brackets. A quasilinear increase of the frictional forces was observed for increasing amounts of apical and buccal malalignments. *Conclusion*. The Synergy bracket with silicone ligature placed around the inner tie-wings appears to yield the best performance.

## 1. Introduction

During fixed appliance therapy, friction generated at the bracket/wire and wire/ligature interfaces is a critical factor in determining the efficiency of biological tooth movement [1]. Friction is the resistance to motion triggered by tangential movement of an object pushed against another one [2]. Friction may be affected by different factors such as the environmental conditions (i.e., temperature, presence of lubricants, etc.), the surface topography, and the material properties [3]. 

Orthodontic treatment with sliding mechanics involves a relative displacement of wire through bracket slots, and whenever sliding occurs, frictional resistance will arise. During the early alignment phase, when all the teeth move at the same time and the wire slides through 10 brackets and 2 tubes, low levels of friction may be required. For this reason, many studies have proposed different methods to limit frictional forces at the bracket/wire and wire/ligature interfaces, such as loosely tied stainless steel ligatures [4], nonconventional ligature systems [5, 6], self-ligating brackets [7], and low-friction brackets [8]. Self-ligating brackets are ligatureless brackets classified into two main categories: those with a spring clip that presses against the archwire (“active” or “interactive” self-ligating brackets) and those in which the self-ligating clip does not press against the archwire (“passive” self-ligating brackets). Passive self-ligating brackets have been shown to induce less friction during sliding mechanics than active self-ligating brackets, except in the case of undersized archwires [9]. When employing low-friction brackets, thanks to the use of high performance materials, as well as the optimal shape given to the slot and to the ligating systems, reduced friction force values can be obtained [8]. Decreased levels of friction have also been demonstrated with the use of nonconventional elastomeric ligatures on conventional brackets as compared with conventional elastomeric ligatures [5]. 

Many authors [10–22] have measured the friction force released during sliding mechanics with different brackets and ligating systems. Other authors evaluated how the friction changes according to different states of lubrication [6] or different states of corrosion of the brackets materials [23]. Special issues have been devoted to the topic (Seminars in Orthodontics (December 2003, Vol. 9, no. 4)) [3, 24–29]. Most of the previous studies have assessed the frictional force by using a single bracket model coupled with an orthodontic wire sliding at different angles [6, 8, 16, 30, 31]. Tecco et al. [9] evaluated the total friction produced by various bracket-archwire combinations in a testing model that includes 10 aligned brackets and concluded that the use of a 10-bracket model elicits more data than the previous studies [32–35] as well as provides the clinician and the research worker with additional interesting information about the frictional force of the various bracket-archwires combinations. On the basis of this rationale, various studies have been conducted utilizing experimental models that included different brackets located in the appropriate relative positions. Franchi et al. [5] utilized a five-bracket model reproducing the right buccal segment of the maxillary arch to assess the frictional forces produced by different bracket/ligature combinations. They did not simulate any malalignment. On the other hand, Matarese et al. [21] and Cordasco et al. [22] developed an experimental setup with three nonleveled brackets, simulating a fixed level of malalignment of 1 mm. Yeh et al. [36] developed a testing model including three brackets placed at malaligned positions. Henao and Kusy [37, 38] utilized a dental typodont including different teeth with fixed levels of malocclusion. In the present study, we have expanded the above testing models. We increased the number of brackets, as compared to Matarese et al. [21], Cordasco et al. [22], and Yeh et al. [36], while, as compared to Franchi et al. [5] and Henao and Kusy [37, 38], we included the effect of various degrees of apical and buccal malalignments of the canine. The designed setup was utilized to investigate whether the following factors affect the values of friction force during the early alignment phase: (i) degree of malalignment, (ii) diameter of the orthodontic wire, and (iii) bracket/ligature combination. In total, 535 measurements of friction force were carried out. Statistical analyses by three-way ANOVA and Tukey post hoc test were applied to the values obtained. 

## 2. Materials and Methods

### 2.1. Experimental Setup

**Figure 1:**
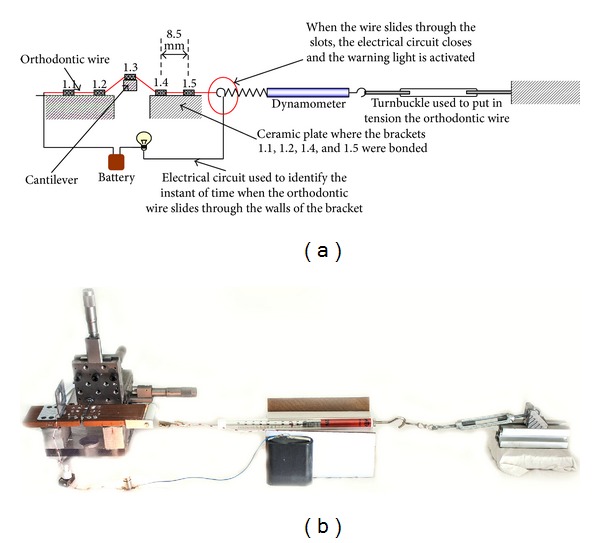
Scheme (a) and picture (b) of the experimental setup used to measure the friction force at the bracket/wire and wire/ligature interfaces.

An experimental setup was designed to measure the friction force generated by five different brackets (Figure 1): three self-ligating brackets, (Damon 3 mx (SDS Ormco, Orange, CA, USA), Smart Clip (3M Unitek, Monrovia, CA, USA), and Time 3 (American Orthodontics, Sheboygon, WI, USA)), one low-friction bracket (Synergy (Rocky Mountain Orthodontics; Denver, CO, USA)), and one conventional stainless steel bracket (control) (Dentsply Maillefer, OK, USA). 

**Figure 2:**
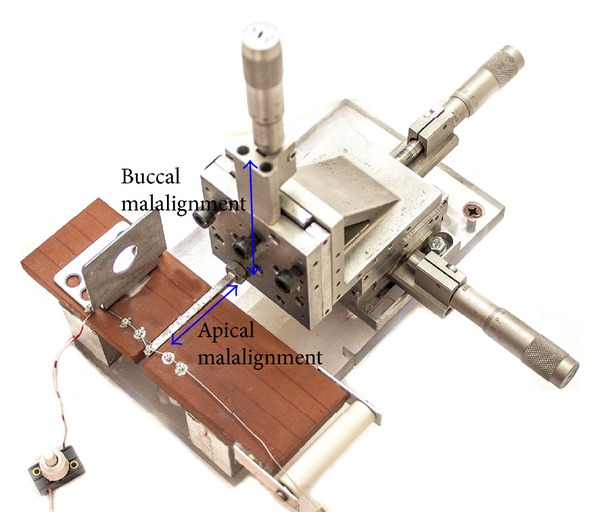
High precision linear translation stage used to reproduce apical and buccal malalignments.

All the tested brackets had a 0.022 in slot; following Franchi et al. [5], the interbracket distance was set equal to 8.5 mm. Ni–Ti 0.014 and 0.016 in (Rocky Mountain Orthodontics; Denver, CO, USA) straight lengths wires were tested. All tests were of brackets for the right buccal segment of the maxillary arch. The brackets 1.1 (central incisor) and 1.2 (lateral incisor) as well as the brackets 1.4 (first premolar) and 1.5 (second premolar) were bonded onto a 60 × 50 × 5 mm ceramic plate (Figure 1(a)) with epoxy resin. Instead, the bracket 1.3 (canine) was bonded with the same resin onto a 0.5 × 0.5 × 65 mm stainless steel cantilever (Figure 1(a)) connected to a high precision linear translation stage (Figure 1(b)). A section of 0.022 × 0.022 in stainless steel wire was used to align the brackets before fixing them. By means of the cantilever, different degrees of buccal and apical malalignment of the canine were reproduced (Figure 2). The end of the orthodontic wire was fastened to a dynamometer hook. Since the values of the measured friction forces changed by more than one order of magnitude, three different dynamometers were used, with different measuring ranges: (a) 3B Scientific GmbH (Hamburg, Germany) U20030, measuring range 0.1 N, accuracy 0.001 N; (b) 3B Scientific GmbH (Hamburg, Germany) U20033, measuring range 2 N, accuracy 0.02 N; (c) WUNDER SA.BI. Srl (Trezzo sull'Adda, Italy), measuring range 150 N, accuracy 0.2 N. An accurate calibration process was conducted on each of the dynamometers by means of a standard set of weights. The accuracy of the proposed experimental setup was found to be comparable to that declared by the dynamometer manufacturers. Only in the case of the 3B Scientific GmbH U20030 dynamometer a 10% greater accuracy was recorded. A turnbuckle connected to the other hook of the dynamometer was activated to put in tension the orthodontic wire. An electrical circuit including a portion of the archwire was used to identify the instant when sliding occurs. When the tensile force acting on the wire becomes greater than the static friction force generated by the brackets, the wire slides through the bracket slots, thus allowing closure of the electrical circuit (Figure 1(a)), which causes a warning light to be activated. The friction force at the bracket/wire and wire/ligature interfaces (BWWLIs) was assumed to be the maximum force value registered by the dynamometer (i.e., immediately before the wire starts to slide). 

The friction generated by the testing unit consisting of wire, brackets, and ligation systems was measured in dry conditions and at room temperature (20°C ± 2°C).

Three different Experimental Campaigns (EC) were conducted.

### 2.2. Identification of the Factors Affecting the Frictional Force Values at the BWWLI

**Figure 3:**
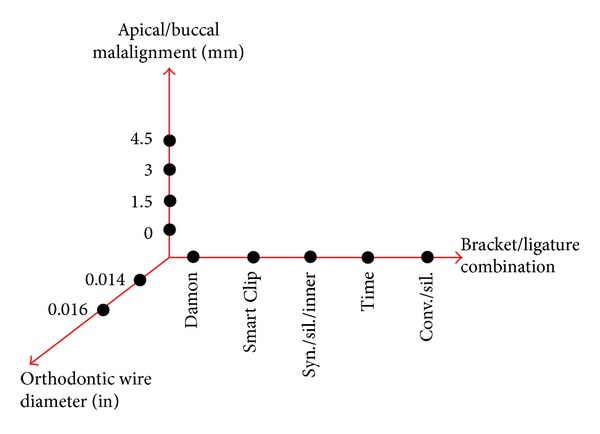
Complete factorial plan designed to identify the factors that have a statistically significant influence on the friction force generated at the bracket/wire and wire/ligature interfaces.

The first and the second experimental campaigns (EC1 and EC2) were aimed at identifying, by three-way Analysis of Variance (ANOVA), the factors that affect the friction force at the BWWLI. A complete factorial plan was drawn up, assessing three factors: (i) degree of the malalignment, (ii) diameter of the orthodontic wire, and (iii) bracket/ligature combination. The null hypothesis *H*_0_ was that factors (i), (ii), and (iii) do not affect the friction force values. *H*_0_ was assumed to hold true for *P*  values ≥ 0.05 (95% confidence interval). For factor (i) four levels were fixed: 0, 1.5, 3, and 4.5 mm; for factor (ii) two levels: 0.014 and 0.016 in, finally, for factor (iii), five levels: Damon, Smart Clip, Synergy with silicone ligature placed around the inner tie-wings, Time, and Conventional with silicone ligature (Figure 3). In total, 4(malalignment)×2(wire_diam⁡)  ×5(bracket_type)×5(repetitions) = 200 experiments were conducted in each experimental campaign, each experiment being repeated five times. The only difference between EC1 and EC2 is that in EC1 the apical malalignment was investigated versus the buccal malalignment in EC2 (Figure 2). A new orthodontic wire was used in each experiment. In EC2 the total number of measurements was 175 because 25 measurements, referring to a malalignment equal to 0.0 mm, had already been carried out in EC1. 

A normal data distribution and equality of variance were not found (Shapiro-Wilk and Levene's tests). A nonparametric test (analysis of variance on ranks with Tukey posthoc test) was therefore used to compare the self-ligating and the low-friction systems with the conventional brackets (control). 

### 2.3. Comparison of Different Ligating Systems for Conventional Brackets

The goal of experimental campaign EC3 was to compare different ligating systems for conventional brackets. Using the experimental setup described above (Figures 1 and 2), three different ligatures made of different materials were tested: stainless steel, conventional elastomeric, and silicone ligatures. The test was conducted only on the stainless steel conventional bracket. The friction force was evaluated for each diameter of the orthodontic wire and for each of the four degrees of malalignment. Since each experiment was repeated five times and both apical and buccal malalignments were investigated in EC3, a total of 3(ligatures)×2(wire_diam⁡)×4(malalignment)×5(repetitions)×2(apical/buccal) = 240 experiments were conducted; since 80 experiments had already been conducted in EC1 and EC2, the total number of measurements carried out in EC3 was equal to 160. 

## 3. Results

**Figure 4:**
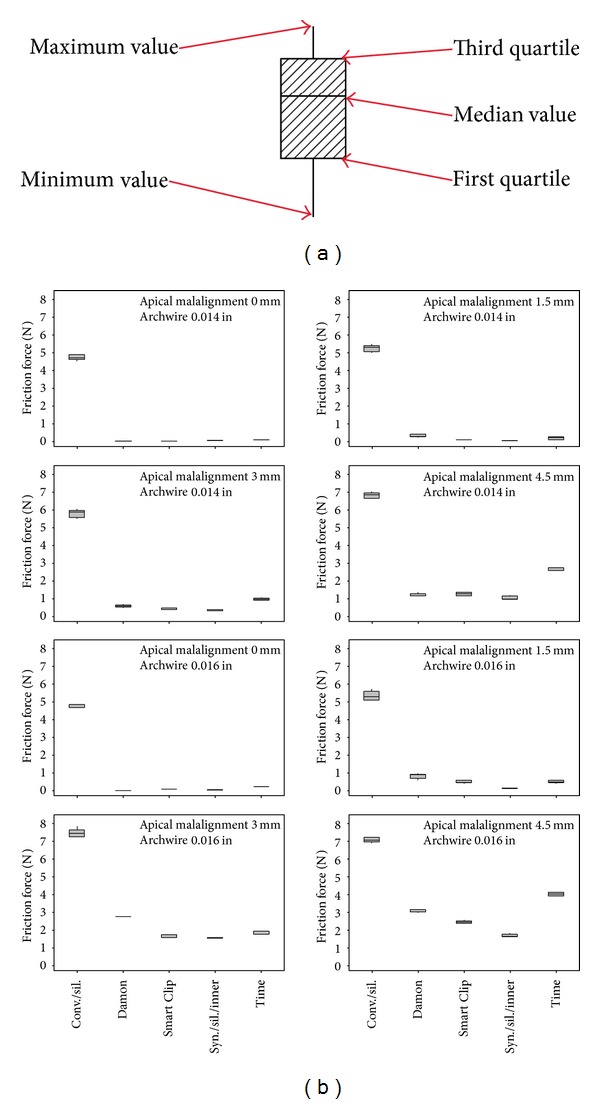
Boxplot of the friction force generated by different brackets for different levels of apical malalignment of the canine and diameters of the orthodontic wire (b). For each of the investigated bracket/ligature combinations, the median value, the first quartile, the third quartile, and the minimum and the maximum values are indicated (a).

**Figure 5:**
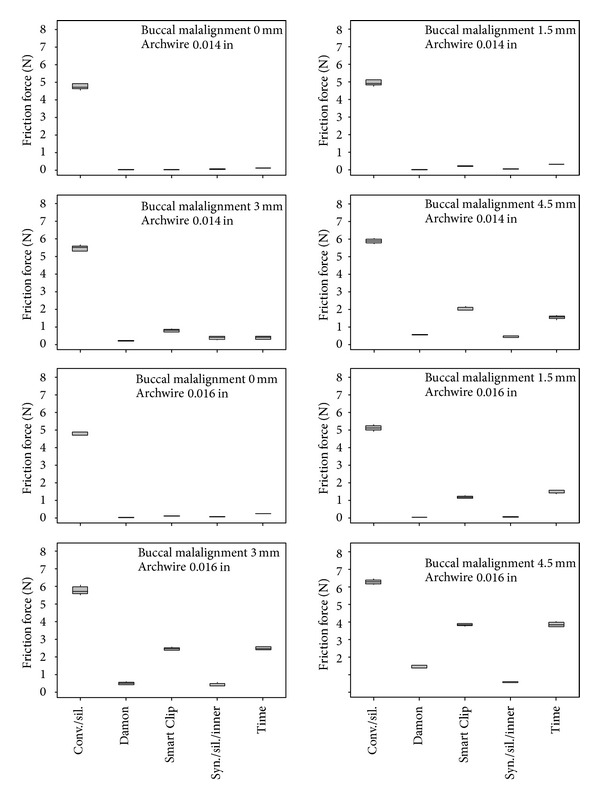
Boxplot of the friction force generated by different brackets for different levels of buccal malalignment of the canine and diameters of the orthodontic wire.

**Figure 6:**
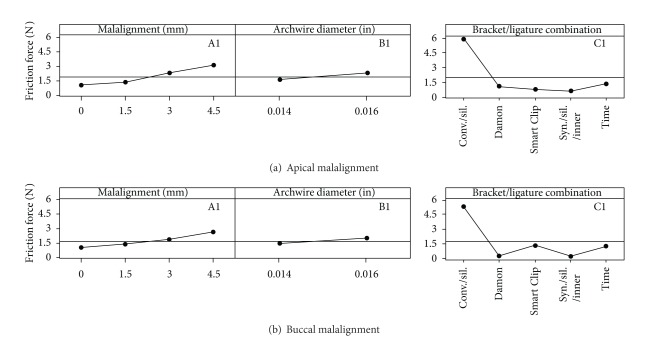
Main effects' plot reporting the values of friction force averaged over the factors (i) degree of malalignment of the canine, (ii) diameter of the orthodontic wire and, (iii) bracket/ligature combination for both the apical (a) and the buccal (b) malalignments. The horizontal line reported in the three diagrams A1, B1, and C1 (apical malalignment) or A2, B2, and C2 (buccal malalignment) represents the average friction force value measured in all the experiments conducted in EC1 (apical malalignment) or in EC2 (buccal malalignment). For example, the value of friction force reported in the diagram A1 for a level of malalignment of 1.5 mm represents the average friction force value measured in all the experiments (carried out in the experimental campaign EC1) with a malalignment of 1.5 mm. Similarly, the friction force value reported in the diagram C1 for the Synergy bracket (silicone ligature placed around the inner tie wings) represents the value averaged over all the measurements carried out on a Synergy bracket in the experimental campaign EC1. All the values reported in the figure can be interpreted in the same way.

Three-way Analysis of Variance revealed that the factors (i) degree of malalignment of the canine, *P*  value = 0, (ii) diameter of the orthodontic wire, *P*  value = 0, and (iii) bracket/ligature combination, *P*  value = 0, certainly do affect the friction force values at the BWWLI, as regards both apical and buccal malalignments. The friction force values that appear to depend most strongly on the specific bracket/wire combination increase with increasing levels of malalignment as well as with increasing values of the orthodontic wire diameter (Figures 4(b) and 5). For each of the investigated bracket/ligature combinations, the median value, the first quartile, the third quartile, and the minimum and maximum values are indicated (Figure 4(a)). The mean friction force values for factors (i), (ii), and (iii) are shown in the main effects plot (Figure 6). 

Table 1 (apical malalignment) and Table 2 (buccal malalignment) show the mean values and standard deviation (computed over the five repetitions of each experiment) and statistical comparisons of friction forces generated by the different bracket/wire combinations for the different archwire diameters and degrees of malalignment. 

## 4. Discussion

In the present study, an experimental setup reproducing the right buccal segment of the maxillary arch was designed to measure the friction force generated by different bracket types and with different degrees of apical and buccal malalignments of the canine. Different combinations of bracket, orthodontic wire, and ligating systems were tested. 

The present study has some limitations. Firstly, apical and buccal malalignments were considered separately (i.e., when the value of apical malalignment was assumed to be different from zero, the level of buccal malalignment was set at zero, and vice versa). Instead, in clinical practice, buccal and apical malalignments are often present simultaneously. As a first approximation, one can reasonably assume that the friction force produced in cases with both apical and buccal malalignments is represented by the vectorial sum of the component of friction force related to both apical and buccal malalignments. Nonetheless, further investigations should be carried out in this field. The friction force was measured in vitro, under dry conditions, at room temperature, and on a simplified testing model. Similar conditions were created in other studies [5, 31]. This study, like any in vitro study, cannot accurately simulate what really happens in clinical situations, where a large number of unpredictable and uncontrollable variables are involved, such as the masticatory forces [39], oral functions [9, 40], different degrees of malocclusion [36–38], and the temperature and moisture conditions [40, 41]. However, after making in vivo and in vitro comparisons, Jost-Brinkmann and Miethke [42] concluded that the friction forces of motionless brackets in laboratory appliances were similar to the forces exerted in clinical appliances [17]. The measurement of the friction force was carried out using dynamometers, unlike in other studies that utilized testing machines with calibrated load cells (e.g., [6, 30, 36, 40]). However, the preliminary calibration procedures conducted on dynamometers demonstrated that the proposed testing model guarantees a comparable accuracy of measurement to that declared by the dynamometers manufacturers and so also comparable to the accuracy achievable with a testing machine. Despite its cumbersome dimensions, the experimental design is easy to set up and replicate and the cost is at least two orders of magnitude smaller than that of a traditional testing machine equipped with a load cell for the measurement of small intensity forces. Because archwires with a fairly small diameter were tested, the frictional forces generated by the self-ligating brackets were measured only in the passive configuration. However, as stated in the Introduction, the aim of the study was to measure the frictional force generated by different brackets during the early alignment phase, when only small diameter wires are allowed. Only Ni-Ti archwires have been tested in the present study. It would be very interesting to observe how the friction changes according to the use of orthodontic wires made of different materials but again, as stated in the Introduction, the objective of the study was to measure the frictional forces in the initial stages of orthodontic treatment, when very flexible archwires like the Ni-Ti wires are appropriate. Only some of the factors influencing the frictional force at BWWLI were considered in this study. Other factors that could certainly play a role of great importance, and will be the object of future studies, include the wire geometry and the bracket width. 

The various levels of malalignment have been simulated by simply activating a screw. Other studies, instead, utilized different testing models with the teeth placed at malaligned positions [36] or a dental typodont including different teeth with various levels of malocclusion [37, 38]. The use of a single testing model that can take on different configurations certainly allows more reliable and accurate measurements to be made. The variables involved in the present study are less numerous than those in others (e.g., the precision with which every experimental model is built, and the precision with which the brackets are bonded on each model), thus achieving a more controllable system response. The changes in the force values measured in the different tests can be regarded as strictly dependent on the variables that were changed before the execution of the experiment and not on other uncontrollable factors. 

Analysis of Variance revealed that all the factors considered, (i) degree of canine malalignment, (ii) diameter of the orthodontic wire, and (iii) bracket/ligature combination, affect (*P*  value = 0) the friction force values in both apical and buccal malalignments. The friction force increased with increasing degrees of canine malalignment and with increasing diameters of the archwire (Figures 6(a) and 6(b)). Similar results have been obtained in previous studies [43–47]. The friction force measured for the self-ligating and the low-friction brackets showed a smaller intensity and variability as compared to the force measured for the conventional stainless steel brackets (Figures 4 and 5). This is true not only for fixed malalignment (as already shown in previous studies [5, 9]) but also for various degrees of malalignment. This result may have a twofold explanation: (i) for small degrees of canine malalignment, the self-ligating and the low-friction brackets behave like a tube that serves only to guide the wire in the correct position; the ligature placed on a conventional bracket, instead, prevents not only micromovements perpendicularly to the wire but also sliding of the wire through the bracket walls; (ii) for larger degrees of malalignment, in addition to the above-described phenomena of friction, other components of resistance to sliding arise such as phenomena like binding and notching of the wire against the corner of the bracket. These phenomena that are circumscribed, sudden, and unpredictable occur less frequently in self-ligating and low-friction brackets, where the presence of a flared slot allows better guidance of the wire at the bracket corner. 

For a malalignment equal to 0 mm, the friction measured for the conventional bracket was on average 5-6 times higher than the friction measured for the other brackets (Figures 4 and 5). For larger degrees of malalignment, instead, this difference was smaller although the force registered for the conventional bracket was always about twice as large as the force measured for the self-ligating and low-friction brackets. Tukey post hoc test showed that for every degree of apical (Table 1) and buccal (Table 2) malalignments, there were statistically significant differences between the friction force generated by conventional stainless steel brackets (control) and the force generated by the others (self-ligating and low-friction brackets). Interestingly, the frictional forces measured for the conventional bracket and for buccal malalignment were, on average, smaller than those registered in the case of apical malalignment. This can be explained by the fact that, for apical malalignment, the contact between the wire and the ligature plays a more important role than that for buccal malalignment. 

Among the self-ligating and low-friction brackets, the Synergy, with a silicone ligature placed around the inner tie wings, yielded the lowest friction force values (Figure 6). In the case of apical malalignment, the Synergy bracket was followed, in terms of friction force, by the Smart Clip, Damon, and Time, and, for buccal malalignment, by the Damon, Smart Clip, and Time (Figure 6). Similar results have been reported by Tecco et al. [9], who found that for Ni-Ti 0.014 and 0.016 in wires, the frictional forces generated by the Damon are slightly smaller than those produced by the Time. They found this result for a null malalignment, while we observed that this same behaviour can be seen for all the malalignment levels investigated. Increasing the wire diameter from 0.014 in to 0.016 in led to friction forces that were, on average, 20% higher (Figure 6). This occurs because as the diameter of the wire increases, the extension of the contact area between the wire and the bracket increases as well as between the wire and the ligature, consequently increasing the total friction force. The parallel increase of the frictional forces with the wire diameter was slightly larger in the case of apical as compared to buccal malalignment. 

**Figure 7:**
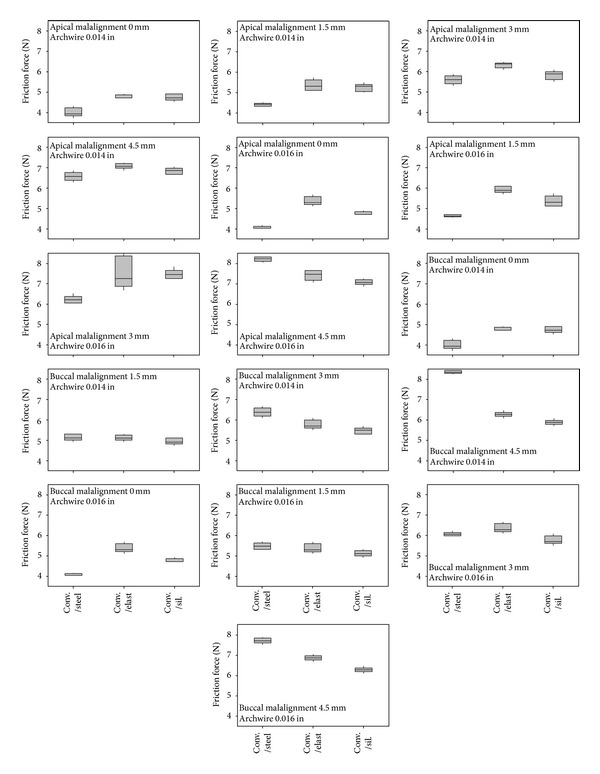
Friction force measured on a conventional stainless steel bracket for different ligation systems (ligature made of different materials: steel, conventional elastomeric, and silicone), orthodontic wire diameters, and amounts of canine malalignment.

The tests carried out in the experimental campaign EC3 demonstrated, in agreement with Iwasaki et al. [48], that stainless steel ligatures produce variable ligation forces (Figure 7). In both apical and buccal malalignments, the lowest friction force values were recorded for the silicone ligature. Again, due to binding and notching phenomena, increasing friction force values were obtained with increasing the degrees of apical and buccal malalignments (Figure 7). On average, the silicone ligature yielded a friction force that was about 10% smaller than the force produced by the conventional elastomeric ligature. 

## 5. Conclusions

1. All the investigated factors, (i) degree of canine malalignment, (ii) diameter of the orthodontic wire, and (iii) bracket/ligature combination, affect the “friction force” response.
2. The conventional brackets exhibited a frictional force 5-6 times higher than the force generated by the other brackets. Despite very high standard deviation values for the conventional brackets at every degree of apical and buccal malalignments, there were statistically significant differences between the friction force generated by these brackets as compared to the others (self-ligating and low-friction brackets).
3. Among the self-ligating and low-friction brackets, the Synergy with a silicone ligature placed around the inner tie wings yielded the lowest friction force values at all the degrees of malalignment tested in this study.

## Figures and Tables

**Table 1:** Average values (computed over the five repetitions of each experiment) and standard deviation of the friction forces measured in experimental campaign EC1 (apical malalignment). Statistical comparisons (Tukey post hoc test, control group 5: conventional stainless steel bracket) are shown in the last column.

	Damon (1)	Smart Clip (2)	Syn/inner (3)	Time (4)	Conv./sil. (5)	Comparison versus group (5)
	(N)	(N)	(N)	(N)		
0.014 (in)–0.0 (mm)	0.0010 ± 0.0007	0.0012 ± 0.0004	0.0392 ± 0.0098	0.0916 ± 0.0015	4.7480 ± 0.1600	1.2.3.4.
0.014 (in)–1.5 (mm)	0.3139 ± 0.0820	0.0806 ± 0.0019	0.0258 ± 0.0043	0.1766 ± 0.0820	5.2385 ± 0.1900	1.2.3.4.
0.014 (in)–3.0 (mm)	0.5886 ± 0.0690	0.4513 ± 0.0540	0.3480 ± 0.0410	0.9810 ± 0.0690	5.8075 ± 0.2200	1.2.3.4.
0.014 (in)–4.5 (mm)	1.2164 ± 0.0880	1.2753 ± 0.0980	1.0540 ± 0.0950	2.6879 ± 0.0880	6.8277 ± 0.1600	1.2.3.4.
0.016 (in)–0.0 (mm)	0.0070 ± 0.0007	0.0840 ± 0.0012	0.0490 ± 0.0069	0.2240 ± 0.0089	4.7676 ± 0.0880	1.2.3.4.
0.016 (in)–1.5 (mm)	0.8240 ± 0.1500	0.5101 ± 0.0820	0.1420 ± 0.0150	0.5101 ± 0.0820	5.34804 ± 0.28	1.2.3.4.
0.016 (in)–3.0 (mm)	2.7468 ± 0.0000	1.6284 ± 0.088	1.5530 ± 0.0084	1.8246 ± 0.0880	7.4556 ± 0.2400	1.2.3.4.
0.016 (in)–4.5 (mm)	3.0607 ± 0.0820	2.4328 ± 0.0820	1.6800 ± 0.0760	4.0221 ± 0.0980	7.0828 ± 0.1500	1.2.3.4.

**Table 2:** Average values (computed over the five repetitions of each experiment) and standard deviation of the friction forces measured in experimental campaign EC2 (buccal malalignment). Statistical comparisons (Tukey post hoc test, control group 5: conventional stainless steel bracket) are shown in the last column.

	Damon (1)	Smart clip (2)	Syn/inner (3)	Time (4)	Conv./sil. (5)	Comparison versus group (5)
	(N)	(N)	(N)	(N)	(N)	
0.014 (in)–0.0 (mm)	0.0010 ± 0.0007	0.0012 ± 0.0004	0.0392 ± 0.0098	0.0916 ± 0.0015	4.7480 ± 0.1600	1.2.3.4.
0.014 (in)–1.5 (mm)	0.0170 ± 0.0007	0.2040 ± 0.0260	0.0275 ± 0.0082	0.3080 ± 0.0180	4.9442 ± 0.1640	1.2.3.4.
0.014 (in)–3.0 (mm)	0.1900 ± 0.007	0.7652 ± 0.0820	0.3532 ± 0.1120	0.3728 ± 0.0820	5.4544 ± 0.1640	1.2.3.4.
0.014 (in)–4.5 (mm)	0.5480 ± 0.0080	2.2021 ± 0.0880	0.4313 ± 0.0530	1.5499 ± 0.1070	5.8860 ± 0.1380	1.2.3.4.
0.016 (in)–0.0 (mm)	0.0070 ± 0.0007	0.0840 ± 0.0012	0.0490 ± 0.0069	0.2240 ± 0.0089	4.7676 ± 0.088	1.2.3.4.
0.016 (in)–1.5 (mm)	0.0238 ± 0.0008	1.1772 ± 0.0690	0.0451 ± 0.0112	1.5107 ± 0.0880	5.1208 ± 0.1460	1.2.3.4.
0.016 (in)–3.0 (mm)	0.4708 ± 0.0820	2.4329 ± 0.0820	0.3940 ± 0.1160	2.4721 ± 0.0820	5.7683 ± 0.2240	1.2.3.4.
0.016 (in)–4.5 (mm)	1.4323 ± 0.0880	3.8455 ± 0.0820	0.5490 ± 0.0313	3.8455 ± 0.1280	6.2784 ± 0.1380	1.2.3.4.

## References

[1] Frank CA, Nikolai RJ (1980). A comparative study of frictional resistances between orthodontic bracket and arch wire. *American Journal of Orthodontics*.

[2] Cacciafesta V, Sfondrini MF, Ricciardi A, Scribante A, Klersy C, Auricchio F (2003). Evaluation of friction of stainless steel and esthetic self-ligating brackets in various bracket-archwire combinations. *American Journal of Orthodontics and Dentofacial Orthopedics*.

[3] Rossouw PE (2003). Friction: an overview. *Seminars in Orthodontics*.

[4] Hain M, Dhopatkar A, Rock P (2003). The effect of ligation method on friction in sliding mechanics. *American Journal of Orthodontics and Dentofacial Orthopedics*.

[5] Franchi L, Baccetti T, Camporesi M, Barbato E (2008). Forces released during sliding mechanics with passive self-ligating brackets or nonconventional elastomeric ligatures. *American Journal of Orthodontics and Dentofacial Orthopedics*.

[6] Thorstenson GA, Kusy RP (2003). Effects of ligation type and method on the resistance to sliding of novel orthodontic brackets with second-order angulation in the dry and wet states. *The Angle Orthodontist*.

[7] Kim TK, Kim KD, Baek SH (2008). Comparison of frictional forces during the initial leveling stage in various combinations of self-ligating brackets and archwires with a custom-designed typodont system. *American Journal of Orthodontics and Dentofacial Orthopedics*.

[8] Redlich M, Mayer Y, Harari D, Lewinstein I (2003). In vitro study of frictional forces during sliding mechanics of "reduced-friction" brackets. *American Journal of Orthodontics and Dentofacial Orthopedics*.

[9] Tecco S, Festa F, Caputi S, Traini T, Di Iorio D, D’Attilio M (2005). Friction of conventional and self-ligating brackets using a 10 bracket model. *The Angle Orthodontist*.

[10] Tidy DC (1989). Frictional forces in fixed appliances. *American Journal of Orthodontics and Dentofacial Orthopedics*.

[11] Barlow M, Kula K (2008). Factors influencing efficiency of sliding mechanics to close extraction space: a systematic review. *Orthodontics and Craniofacial Research*.

[12] Burrow SJ (2009). Friction and resistance to sliding in orthodontics: a critical review. *American Journal of Orthodontics and Dentofacial Orthopedics*.

[13] Ehsani S, Mandich MA, El-Bialy TH, Flores-Mir C (2009). Frictional resistance in self-ligating orthodontic brackets and conventionally ligated brackets a systematic review. *The Angle Orthodontist*.

[14] Nishio C, da Motta AFJ, Elias CN, Mucha JN (2004). In vitro evaluation of frictional forces between archwires and ceramic brackets. *American Journal of Orthodontics and Dentofacial Orthopedics*.

[15] Correia Lima VN, Coimbra Rodrigues MER, D’agostini Derech C, de Oliveira Ruellas AC (2010). Frictional forces in stainless steel and plastic brackets using four types of wire ligation. *Dental Press Journal of Orthodontics*.

[16] Guerrero AP, Filho OG, Tanaka O, Camargo ES, Vieira S (2010). Evaluation of frictional forces between ceramic brackets and archwires of different alloys compared with metal brackets. *Brazilian Oral Research*.

[17] Ribeiro Pacheco M, Douglas Oliveira D, Smith Neto P, Correa Jansen W (2011). Evaluation of friction in self-ligating brackets subjected to sliding mechanics: an in vitro study. *Dental Press Journal of Orthodontics*.

[18] Cacciafesta V, Sfondrini MF, Scribante A, Klersy C, Auricchio F (2003). Evaluation of friction of conventional and metal-insert ceramic brackets in various bracket-archwire combinations. *American Journal of Orthodontics and Dentofacial Orthopedics*.

[19] Doshi UH, Bhad-Patil WA (2011). Static frictional force and surface roughness of various bracket and wire combinations. *American Journal of Orthodontics and Dentofacial Orthopedics*.

[20] Reznikov N, Har-Zion G, Barkana I, Abed Y, Redlich M (2010). Measurement of friction forces between stainless steel wires and "reduced-friction" self-ligating brackets. *American Journal of Orthodontics and Dentofacial Orthopedics*.

[21] Matarese G, Nucera R, Militi A (2008). Evaluation of frictional forces during dental alignment: an experimental model with 3 nonleveled brackets. *American Journal of Orthodontics and Dentofacial Orthopedics*.

[22] Cordasco G, Farronato G, Festa F, Nucera R, Parazzoli E, Grossi GB (2009). In vitro evaluation of the frictional forces between brackets and archwire with three passive self-ligating brackets. *European Journal of Orthodontics*.

[23] Kao CT, Guo JU, Huang TH (2011). Comparison of friction force between corroded and noncorroded titanium nitride plating of metal brackets. *American Journal of Orthodontics and Dentofacial Orthopedics*.

[24] Rossouw PE, Kamelchuk LS, Kusy RP (2003). A fundamental review of variables associated with low velocity frictional dynamics. *Seminars in Orthodontics*.

[25] Mendes K, Rossouw PE (2003). Friction: validation of manufacturer’s claim. *Seminars in Orthodontics*.

[26] Kamelchuk LS, Rossouw PE (2003). Development of a laboratory model to test kinetic orthodontic friction. *Seminars in Orthodontics*.

[27] Smith DV, Rossouw PE, Watson P (2003). Quantified simulation of canine retraction: evaluation of frictional resistance. *Seminars in Orthodontics*.

[28] Kusy RP, Whitley JQ (2003). Influence of fluid media on the frictional coefficients in orthodontic sliding. *Seminars in Orthodontics*.

[29] Iwasaki LR, Beatty MW, Nickel JC (2003). Friction and orthodontic mechanics: clinical studies of moment and ligation effects. *Seminars in Orthodontics*.

[30] Voudouris JC, Schismenos C, Lackovic K, Kuftinec MM (2010). Self-ligation esthetic brackets with low frictional resistance. *The Angle Orthodontist*.

[31] Thorstenson GA, Kusy RP (2002). Effect of archwire size and material on the resistance to sliding of self-ligating brackets with second-order angulation in the dry state. *American Journal of Orthodontics and Dentofacial Orthopedics*.

[32] Andreasen GF, Quevedo FR (1970). Evaluation of friction forces in the 0.022 x 0.028 edgewise bracket in vitro. *Journal of Biomechanics*.

[33] Braun S, Bluestein M, Moore BK, Benson G (1999). Friction in perspective. *American Journal of Orthodontics and Dentofacial Orthopedics*.

[34] Khambay B, Millett D, McHugh S (2004). Evaluation of methods of archwire ligation on frictional resistance. *European Journal of Orthodontics*.

[35] Khambay B, Millett D, McHugh S (2005). Archwire seating forces produced by different ligation methods and their effect on frictional resistance. *European Journal of Orthodontics*.

[36] Yeh CL, Kusnoto B, Viana G, Evans CA, Drummond JL (2007). In-vitro evaluation of frictional resistance between brackets with passive-ligation designs. *American Journal of Orthodontics and Dentofacial Orthopedics*.

[37] Henao SP, Kusy RP (2004). Evaluation of the frictional resistance of conventional and self-ligating bracket designs using standardized archwires and dental typodonts. *The Angle Orthodontist*.

[38] Henao SP, Kusy RP (2005). Frictional evaluations of dental typodont models using four self-ligating designs and a conventional design. *The Angle Orthodontist*.

[39] Griffiths HS, Sherriff M, Ireland AJ (2005). Resistance to sliding with 3 types of elastomeric modules. *American Journal of Orthodontics and Dentofacial Orthopedics*.

[40] Tecco S, Di Iorio D, Cordasco G, Verrocchi I, Festa F (2007). An in vitro investigation of the influence of self-ligating brackets, low friction ligatures, and archwire on frictional resistance. *European Journal of Orthodontics*.

[41] Reicheneder CA, Baumert U, Gedrange T, Proff P, Faltermeier A, Muessig D (2007). Frictional properties of aesthetic brackets. *European Journal of Orthodontics*.

[42] Jost-Brinkmann P, Miethke RR (1991). Einfluß der physiologischen zahnbeweglichkeit auf die friktion zwischen bracket und bogen. *Fortschritte der Kieferorthopädie*.

[43] Kapila S, Angolkar PV, Duncanson MG, Nanda RS (1990). Evaluation of friction between edgewise stainless steel brackets and orthodontic wires of four alloys. *American Journal of Orthodontics and Dentofacial Orthopedics*.

[44] Tanne K, Matsubara S, Shibaguchi T, Sakuda M (1991). Wire friction from ceramic brackets during simulated canine retraction. *The Angle Orthodontist*.

[45] Dickson JA, Jones SP, Davies EH (1994). A comparison of the frictional characteristics of five initial alignment wires and stainless steel brackets at three bracket to wire angulations—an in vitro study. *British Journal of Orthodontics*.

[46] Sims AP, Waters NE, Birnie DJ (1994). A comparison of the forces required to produce tooth movement ex vivo through three types of pre-adjusted brackets when subjected to determined tip or torque values. *British Journal of Orthodontics*.

[47] Read-Ward GE, Jones SP, Davies EH (1997). A comparison of self-ligating and conventional orthodontic bracket systems. *British Journal of Orthodontics*.

[48] Iwasaki LR, Beatty MW, Randall CJ, Nickel JC (2003). Clinical ligation forces and intraoral friction during sliding on a stainless steel archwire. *American Journal of Orthodontics and Dentofacial Orthopedics*.

